# Would you consider donating your left-over embryos to treat Parkinson’s disease? Interviews with individuals that underwent IVF in Sweden

**DOI:** 10.1186/s12910-022-00864-y

**Published:** 2022-12-03

**Authors:** Karin Schölin Bywall, Jan Holte, Thomas Brodin, Mats Hansson, Jennifer Drevin

**Affiliations:** 1 Centre for Research Ethics and Bioethics, Uppsala University, Box 564, 751 22 Uppsala, Sweden; 2 Carl Von Linnékliniken, Uppsala Science Park, 751 83 Uppsala, Sweden; 3 School of Health, Care and Social Welfare, Division of Health and Welfare Technology, Mälardalen University, Västerås, Sweden

**Keywords:** Cryopreserved embryos, Ethical aspects, Human embryonic stem cells, Parkinson’s disease, Qualitative research

## Abstract

**Background:**

Parkinson’s disease (PD) has been considered to be one of the most promising target diseases for forthcoming cell-based therapy. The aim of this study is to explore the views of individuals with cryopreserved embryos on using human embryonic stem cells for treating PD.

**Methods:**

The study was performed as a qualitative, semi-structured interview study in June–October 2020. Participants were recruited at a private fertility clinic located in one of the larger Swedish cities. The clinic provides both publicly financed and privately financed IVF-treatments. All interviews were performed by telephone and analyzed using thematic content analysis. Five main categories emerged from 27 sub-categories.

**Results:**

In total, 18 interviews were performed with 22 individuals, as either a couple (n = 16) or separately (n = 6). Participants had different views on what a cryopreserved embryo is. Some participants addressed cryopreserved embryos as ‘a lump of cells’, and some in terms of their ‘unborn child’. Conditions for donation of cryopreserved embryos for cell-based treatment in PD were: not losing control of what is happening to the embryo, that donating must be voluntary and based on informed consent with time for reflection, that reimbursement, equality and transparency.

**Conclusions:**

Using cryopreserved embryos to treat PD is associated with fundamental ethical and practical issues. This study shows that IVF couples with left-over embryos may be supportive but there is a need for future research to assess people’s views on using cryopreserved embryos for cell-based treatment in PD on a more aggregated level.

## Background

Stem cell research has raised expectations due to the capacity to differentiate cells into a broad range of cell types [1]. Several initiatives on stem cells are forthcoming in different areas; including treating genetic disorders and generating new stem cell-derived human tissues and biomaterials for use in pharmacogenomics and in regenerative medicine [2, 3]. A major breakthrough in stem cell research came in 1994 when researchers derived the first embryonic stem cells from primate embryos [4]. The derivation of the first human Embryonic Stem Cells (hESC) came four years later using 36 donated left-over embryos from patients who underwent IVF. Five hESC lines were derived [5]. These and other stem cell lines carry a potential for regenerative medical treatment for patients with PD [6]. Currently, researchers explore the potential for cell-based therapy by deriving hESC to alleviate debilitating neurodegenerative disorders like PD where predominantly dopamine producing neurons are lost [7]. Autologous transplantation of human induced pluripotent stem cells (iPS-cells), derived from umbilical cord blood or adult tissues may also be a future treatment alternative [8].

PD is a neurodegenerative disorder that affects about 0.3% of the general population [9]. As the population ages, the burden on society increases both in economic terms and in quality of life for patients and their families [10]. The adult brain has a limited capacity to repair, and new neurons are not generated after injury and disease [11]. Currently available pharmacological or surgical treatments may only slow the progressive course of the disease [12]. Motor impairments in PD result from loss of midbrain dopamine neurons. Cell-based therapy, including use of hESC, may be a viable treatment option for restoring dopamine production [5, 6].

The generation of hESCs requires fertilized eggs from donors and implies destruction of early embryos [13], something that has raised ethical and legal concerns [14, 15]. Those who believe that human embryos are subjects with rights are against the destruction of embryos for research, whereas those who view the embryo as too undeveloped to have a moral status generally permit this research. Research is limited in exploring stakeholders' views on embryo donation for treating PD. Donors of left-over cryopreserved embryos are one of the main stakeholder groups in the discussion and it is therefore important to explore their views. The aim of this study is to explore the views of individuals with left-over cryopreserved embryos regarding using human embryonic stem cells for treatment of PD.

## Methods

### Participants

Participants were recruited at a private fertility clinic located in one of the larger Swedish cities. Couples having cryopreserved their embryos during the period 2017–2020 were asked for participation. In order not to put additional psychological stress on couples only those who had experienced a successful IVF treatment were asked to participate, 140 couples were approached by e-mail. Twenty-six persons responded and provided informed consent. Four individuals withdraw before being interviewed. The characteristics of the remaining 22 participants are presented in Table 1.

**Table 1:** The characteristics of the participants (n = 22)

	*n*
*Participation*	
Interviewed as a couple	16
Interviewed separately	6
*Gender*	
Female	14
Male	8
*Age (mean)*	34.7
*Country of birth*	
Sweden	18
Other	4
*Completed level of education*	
Upper secondary school	2
College / University	20
*Embryos cryopreserved since…*	
2017	1
2018	14
2019	7
*Have become parents after undergoing IVF/ICSI treatment*	
Yes	22

### Data collection

The interviews were performed in June–October 2020. All interviews were performed by telephone due to the covid-19 pandemic. When both partners in a relationship accepted participation, they could choose between individual interviews or being interviewed together with their partner. In total, 18 interviews were performed with 22 persons. The participants responded to a brief questionnaire concerning their characteristics before participating (Table 1). Participants were also asked to read some brief information about stem cells, embryonic stem cells and iPS-cells before the interview, to introduce them to the subject. All interviews were performed by the author JD.

### Analyses

The interviews were transcribed verbatim. The interviews were analysed by KSB and JD using thematic content analysis [16]. We followed the COREQ (COnsolidated criteria for REporting Qualitative research) Checklist. The interviews were read by KSB and JD with one interview openly coded (i.e., writing short phrases that sum up meaningful content of a text segment). This was done separately by both researchers. The remaining interviews were analysed by KSB. Differences in coding were discussed and agreed upon. In total, 322 open codes were extracted from the interviews. A shorter list of codes (n = 107) was compiled by merging overlapping or similar codes. Main categories (n = 5) were derived from the codes and sub-categories (n = 26) by further refinement to conclude the final coding framework. Key findings were reported under each main category in the result section, using appropriate quotes to illustrate those findings.

## Results

### Respondents

In total, 22 participants were interviewed, either as a couple (n = 16) or separately (n = 6). Most were female (64%) and born in Sweden (82%), the mean age was 34 years (Table 1).

### Interviews

The thematic content analysis revealed five main categories that emerged from 26 sub-categories: individuals’ views on cryopreserved embryos, general view on donation of cryopreserved embryos, attitudes regarding donating cryopreserved embryos for cell-based treatment in Parkinson’s disease, conditions for donating and fears. These main categories are presented and illustrated using quotes from the interviews below (Table 2).

**Table 2:** Final coding framework

Codes	Sub-categories	Main categories
Potential baby	A potential child	Individuals' views on cryopreserved embryos
Cells are beginning of life		
Cell lump	Not a child	
Not a baby		
Missing a heart		
Not all embryos become children		
Not like any cell	Special	
Higher value		
Moral uncertainty	Moral obligations	
A moral limit		
Does not feel natural		
Help others	Donation can benefit sick people	General view on donation of cryopreserved embryos
Help sick		
Better care		
Good purposes		
Ability to support patients		
Gives hope	Donors' feelings	
It feels good to help others		
Regret		
Unethical experiments	Ethics in donation	
Ethically okay		
Unethical to enhance humans		
Support good causes	Values in donation	
Want to do good		
Help as many as possible	Maximise utility in donation	
Help those with highest needs		
Benefit the most people		
Prioritise severe diseases	Prioritisation in donation	
Not for self-inflicted conditions		
Prioritise public diseases		
Not in the beauty industry		
Only for diseases with no cure		
Relieve health care system	Benefit for society	
Support health care professionals		
Contribute to research		
Contribute to society		
Reducing costs for health care		
Better to use then throw away	Positive to donate to PD	Attitudes regarding donating cryopreserved embryos for cell-based treatment in PD
Cryopreserved embryos can result in something positive		
Someone close with PD		
If no more children		
Contribute to Parkinson treatment		
Finding new treatment alternatives		
Parkinson patients suffer		
Wrong to use embryos for PD	Negative to donate to PD	
Wrong to do research on embryos		
Taking a life		
Wrong to use embryos in medications		
No alternative is okay		
Mixed feelings	Unsure to donate to PD	
May be needing the embryos		
Ambivalent		
Different opinions		
Need more information		
Have not seen PD upfront		
Not ready to make a decision		
Need more knowledge		
Feels weird		
Trade-offs	Balancing benefits and risks	
Increased quality of life		
Important with low risks and high benefit		
Safe treatment		
Losing control	Not losing control	Conditions for donating
Pharmaceutical companies		
Taking care of embryos		
In safe hands		
Can create mistrust	Being asked to donate	
Instead of throwing them away		
Asking when no longer needed		
Asking anytime		
Asking when storage time is over		
It should be up to the couple to donate	Autonomy	
The use of the embryo should be up to the couple		
The couples need to feel that they make the right decision		
Only want to donate to institutes with good values		
Couples need to know where the embryo will go	Informed consent	
It should be up to the researchers to decide once given informed consent		
Research on embryos requires informed consent		
Informed on what disease the embryo will go to		
Not necessary with informed consent in medical treatment		
Informing the donor even if the embryo is not used		
Important with reflection	Reflection	
Weighing different options		
Proper reimbursement to donors	Reimbursement	
Not only for rich people	Equality	
Transparency to public	Transparency	
Not for healthy individuals	Future development	Fears
Lack of autonomy		
Use of donated embryos in other disease areas		
Moral obligations		
Need to limit misconduct of donated embryos	Misconduct of donated embryos	
Being utilized by pharmaceutical companies		
Ignoring the purpose stated in donors' information		
Not supporting donation for some purposes		
Not for human enhancement		
Researchers can get good attention from society	Researchers own interests	
Researchers may have their own interests		
Big profit	Pharmaceutical companies' interests	
Wrong with to big profit margins		
It does not matter where the profit goes		
Accepting profit when necessary		
Profit may be the main interest		
Okay with some profit		
Profit should go to development of new medicines		
Not okay that pharmaceutical companies make profit on embryos		

#### Individuals’ views on the status of cryopreserved embryos

Participants had different views of what a five-day cryopreserved embryo meant to them. Sometimes respondents expressed that the cryopreserved embryos were potential children, imagining them to be ‘unborn’ siblings to their living children:

P12: Actually, a child who is, sort of, one of our children, or potential children or is it just, no I do not know, so I would not have the same feelings for a blood test for example.

Others expressed themselves in more general terms. Sometimes the word ‘cell lump’ came up and someone said that this is not a baby because there is no beating heart:

P15: When I think a little more about it, maybe I feel even more certain that the advantages would be greater than the disadvantages, that it is just an embryo that I only have in the freezer that is five days, that it is not so much. It is not a heart that beats and the whole track, that it is still very young, that it is more like a lump of cells.

The cryopreserved embryo was also mentioned as special with a higher value compared to other cells. This also came along with moral responsibilities and boundaries according to some respondents:

P15: it's the whole philosophical discussion about what a human being really is and when does it start to become a life and what to use an embryo for, it makes sense to sort of use it for only one such thing out of a more like what to say moral gaze, position.

#### General views on donation of cryopreserved embryos

Donation of cryopreserved embryos was mentioned as a good action to give hope and help sick people in need:

P11: I see it as if the embryos are not to be used anyway, if the couple has chosen not to have more children or choose to continue with them, so I think it is positive that they can be used for anything else that could be positive for other diseases.

Some were positive to using embryos for any kind of medical treatment, underlining that the donating couple should decide whether to donate or not:

P14: My summary view is that I am positive about donating embryos, but then in a general view it is still up to the couple if they are ready to throw them away, donate them or donate them to another couple or something like that if it was possible. I am still positive that regardless of disease, that you can research other diseases than just Parkinson's and also medical treatments and so on, I am positive about that. Then it is still an ethical dilemma, I think this with donation and at least ethical dilemma for me like that, to donate to another couple and how to approach it and how to think and so.

Donation of embryos in order to cure disease was mentioned as a good cause, with those in greatest medical being prioritized:

P1: but, also that... you think as rationally what is the best. Which choice will be the best for most people like which, well I do not know that, it is important for you that as many as possible benefit from it.

#### Attitudes regarding donating cryopreserved embryos for cell-based treatment in Parkinson’s disease

Participants’ attitudes differed when thinking about donating cryopreserved embryo for treatment of PD. Some said that they were positive to donate for PD because it is better if the cryopreserved embryos are being used for a good cause instead of thrown away:

P3: I think, I only see it as positive thing, that they are donated, because if they are not to be used for anything, regarding what they were intended for from the beginning, it is better that they are used (i.e., for cell-bases treatment in PD) than that they are discarded.

Some wanted to contribute to research and better medications but thought it was wrong to do research on embryos. One person said:

P13: As I said before, that research is important, it is quite clear that if you can help people and find solutions to Parkinson's, for example, it is absolutely important. But using embryos, which is just wrong according to me.

Several participants were uncertain if they would donate their cryopreserved embryos if they got the question. Either they wanted more information on the donation process or they had not made up their mind on whether to use the embryos themselves to conceive a pregnancy or not:

P1: I would not like to, if I were choosing between having another child or donating, then I would not like to give my embryos away. But maybe if I made the choice not to have any more children.

#### Conditions for donating

If donation of cryopreserved embryos was to become legal, it should come with some donor conditions according to the participants. The donor should ‘not lose control’, meaning that they should be able to know in which circumstances it will be used:

P15: If you were to donate your cells, you have no control really, over what is being done. If you give something away like that, you give some kind of consent to use them. You let go of the control to other people and then it is good to know in which framework this can be used.

Being ‘asked about donation’ was an important condition and also a sensitive topic but it should be brought up only after they had decided not to use the embryos for fertility treatment:

P1: That they should ask, but not before we had decided not to use the embryos, they could ask if we would like to donate to research. It would feel intervening if we would get the question before that.

Autonomy, the right to decide if you want to donate or not was mentioned by several respondents as a fundamental concern when donating embryos:

P2: I can think that it is good, that donation of embryos happens and that it exists. While I can also think that certain things are more private matters, do you understand what I mean? I think it is fantastic but, it should not matter if a couple says yes or no.

Giving ‘informed consent’ and informed about what the donation will be used for was mentioned as an unconditional concern for many of the respondents:

P16: I think the most important is to know what the embryo should be used for and that you can be sure that it is not used for other things than what it is intended for. I think that is especially important when it comes to human cells.

Some mentioned ‘reflection’ as a condition because they would need to reflect before making the decision. Some mentioned that they should be ‘reimbursed’ for the donation as they put a lot of money and time in getting the embryos:

P8: As we said at the beginning, that it still feels hard. If you had asked us now, if we would donate an embryo. I am not there in my mind yet. I need to think about this and come to conclude if not to have any more children. I am not really mentally ready to make that decision right now.

Several respondents expressed donating embryos to pharmaceutical companies as a fear. They did not want the company to gain a lot of profit on their struggle to get pregnant. However, some profit was accepted to be able to further develop medical treatments:

P15: They need to be able to make money for there to be an interest in developing such medicines, I think it is an inevitable part of it. But it is clear that they should not make to big profit out of this.

Equality in terms of ‘who is benefiting from the donation’ was also brought up as well as transparency towards the general public:

P1: No, I do not think it is a problem. It will be a problem if they sell the medicine too expensive, that is what I think, or it the treatment only would go to some very rich person and that someone poor would not have the opportunity. I want them to sort of charge a reasonable price for it.

#### Fears

Several fears were brought up during the interviews. Fearing ‘future development’ using embryos to enhance healthy humans or that IVF couples would feel forced to donate even if they did not want to:

P14: You should not ask people to do an IVF just to get the cells, because it is still a difficult thing to go through. You should still be able to ask those who are undergoing IVF, if they have leftover embryos that they will not use. Instead of being thrown away, they can go there.

Misconduct was also feared by the respondents saying that they do not want their cryopreserved embryos to be used for the wrong purposes, like for something not aligning with their values. Someone said that researchers may have their own interests:

P8: There is probably always a question of personal interests for those who do research on life as well as get noticed in the media.

## Discussion

According to Swedish law donation of left-over embryos to help involuntary childless couples is permitted since 1 January 2019 under certain conditions (Genetic Integrity Act 2006:351). Donation for other purposes, such as using human embryonic stem cells for treating PD, is not permitted in Sweden. The use of human embryos for research is comprehensively regulated. Medical research concerning left-over embryos is permitted only if no more than 14 days have passed after the embryo was formed. The aim of this study was to explore the views of individuals with cryopreserved embryos on using human embryonic stem cells for treating PD. The interviews revealed five main categories with under-ordered sub-categories corresponding to this aim.

In summary, participants of the interviews were in general positive to donation for the purpose of using human embryonic stem cells for treating PD, even if they were not certain that they would say yes if they got the question themselves. This finding aligns with previous research that has found that couples that underwent fertility treatment and members of the general public in Sweden would consent to donate their spare embryos for stem cell research rather than letting them be discarded [17, 18]. The participants had different views on what a cryopreserved embryo is, ranging from a lump of cells to their unborn child. The overall impression is that this did not affect their acceptance of using them for medical treatment, but this needs to be assessed with quantitative methods. Other studies indicate that the symbolic representation of an embryo plays a crucial role for deciding about their potential use [19].

Participants underline the importance of leaving the decision to the couples that underwent the fertility treatment. Similar reasoning has also been identified in interviews with couples in Switzerland, where a main finding focused on developing a fair informed choice procedure that included information about the research project [20]. Embryo donators also need to understand that the choice to donate is up to them and that they will not receive any financial compensation.

The participants in this study identified several conditions that need to be kept in mind when setting up procedures and provide guidance to fertility clinics [21]. They feared that commercial interests could be taking over in comparison to helping patients in need of medical treatment.

This study has some limitations that need to be mentioned. For this study, 22 individuals participated out of the 140 couples that were approached by e-mail. Therefore, results of this study should be interpreted with the respect to the low response rate and may not be generalizable for the whole population of individuals that underwent IVF in Sweden.

With regard to the population, participants were only recruited from one IVF clinic and there was an overrepresentation of participants with a high educational level. An important caveat is that we only recruited couples who had already experienced a successful IVF treatment, a fact that is likely to have effect on attitudes. This is a qualitative study aimed at identifying a wide spectrum of concerns and attitudes held by couples. The results of this study may not be generalizable to countries that differ significantly from Sweden in terms of religiosity, funding for IVF, healthcare systems, etc. Future research is needed to assess views on a representative and aggregated level by using quantitative methods.

## Conclusions

Using human embryonic stem cells as a means for medical treatment has a significant potential to relieve suffering and improve quality of life for patients. However, it is also associated with ethical issues. Individuals with left-over cryopreserved embryos were in general positive to donation for the purpose of using human embryonic stem cells for treating PD, but there were important conditions to keep in mind. They wanted to be in control of the decision to donate or not donate, get information about the purpose and have time for reflection.

## Data Availability

The datasets generated and/or analyzed during the current study are not publicly available since it contains sensitive information, but are available from the corresponding author on reasonable request.

## Abbreviations

hESC: Human embryonic stem cells
PD: Parkinson’s disease
IVF: In vitro fertilization
